# Mediastinal hydatid cyst: a case report

**DOI:** 10.1186/1752-1947-7-236

**Published:** 2013-10-07

**Authors:** Yassine Msougar, Oussama Afandi, Nadia Ihfa, Yassine Baiz, Youness Rouiessi, Mohamed Khellouki, Benacer Finech

**Affiliations:** 1 Service de chirurgie viscérale, CHU Mohamed VI, Marrakech, Morocco; 2 Service d’anesthésie et réanimation, CHU Mohamed VI, Marrakech, Morocco

**Keywords:** Hydatid cyst, Mediastinal, Thoracotomy

## Abstract

**Introduction:**

Mediastinal localization of hydatidosis is very rare even in endemic areas. The diagnosis is based on typical clinical and radiological criteria.

**Case presentation:**

We report a case of a mediastinal location of hydatidosis in a 60-year-old Arab man admitted for chest pain. The chest radiograph showed a rounded and homogeneous opacity. Computed tomography showed a right mediastinal cyst, without other thoracic or abdominal sites. Through a posterolateral thoracotomy, we found a cystic mass in the posterior mediastinum. The patient received a cystectomy with medical treatment based on albendazole. He improved a few weeks later.

**Conclusion:**

Mediastinal cysts remain rare, even in endemic countries, which makes initial diagnosis difficult. Our observation shows the importance of keeping this diagnosis in mind when a patient presents with signs of mediastinal compression.

## Introduction

Hydatidosis, or hydatid cyst (HC), represents an epidemic disease that particularly affects North Africa and South America where areas of traditional breeding predominate. The pathogenesis is due to the accidental infestation of a human by a dog’s dejections which contained the *Taenia Echinococcus granulosus*[1]. It affects different organs, but mainly the liver and lung, whereby the blood filters for parasite dissemination. Mediastinal localization is very rare and poses diagnostic and therapeutic problems [2]. We report a case of mediastinal HC localization.

## Case presentation

A 60-year-old Arab man, who had pulmonary HC surgery 20 years ago, attended a first consultation 1 month after the onset of symptoms: chest pain with a dry cough associated with inspiratory dyspnea, all operating in a context of apyrexia and conservation of general status. A physical examination on admission was normal. A chest radiography showed a rounded and homogeneous opacity.

**Figure 1:**
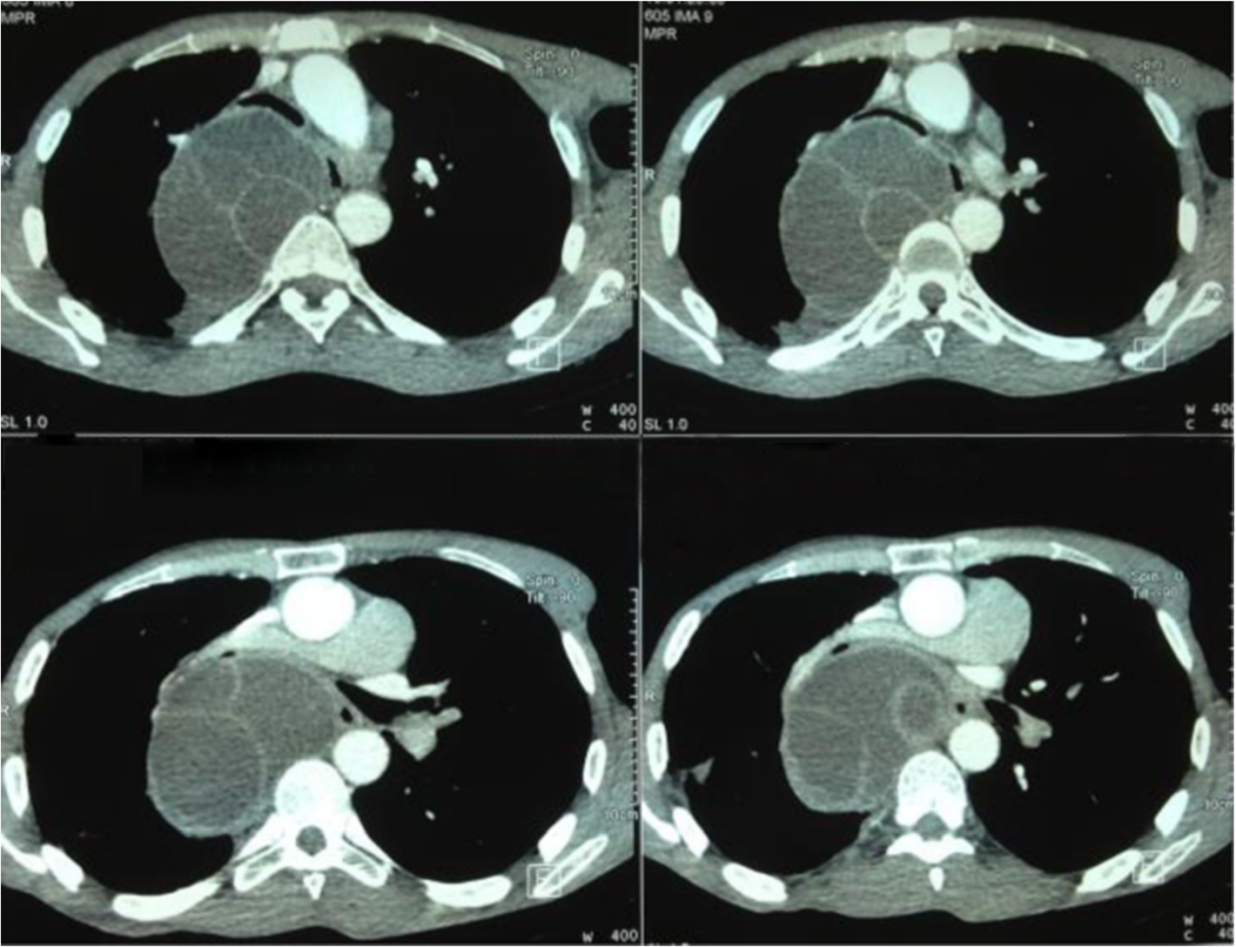
Chest computed tomography after injection of contrast, objectifying a liquid formation in the laterovertebral middle mediastinum with regular contours and fine wall discreetly enhancing by the contrast.

**Figure 2:**
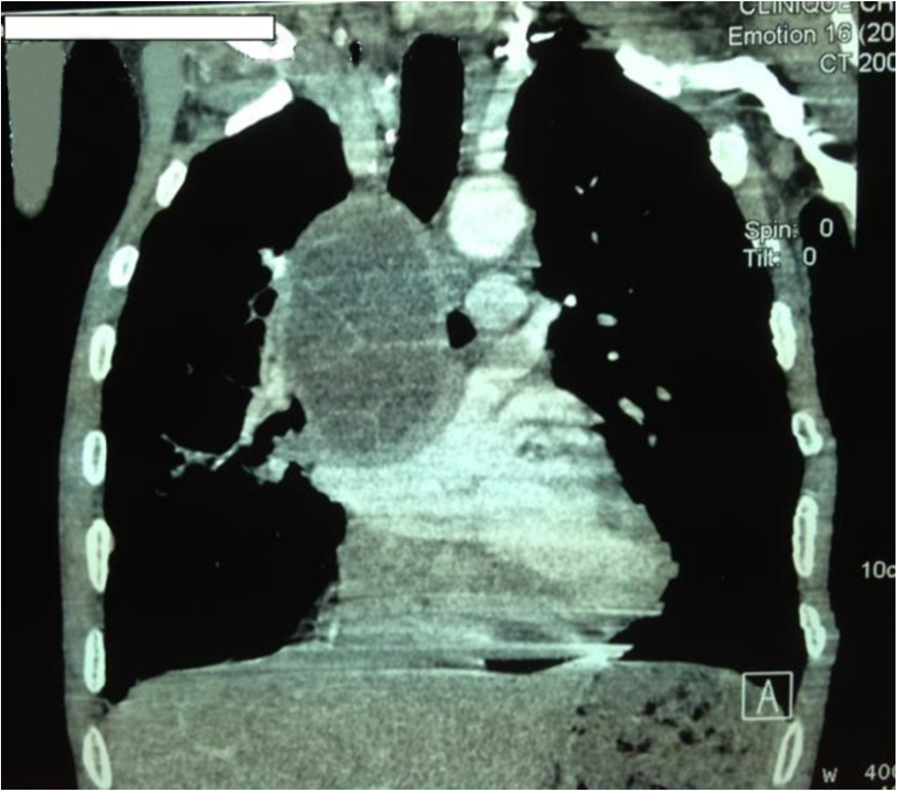
Computed tomography image reconstruction showing the close relationship between the cyst and the bronchus.

A computed tomography (CT) showed, at the middle mediastinum in laterovertebral view, behind the right main bronchus, a liquid formation with well limited contours and regular thin wall containing within cubicles separated by septa. After injection of contrast material, the wall and septa were discreetly enhanced (Figures 1 and 2). We noted the absence of HC intraparenchyma, with the presence of centrilobular and paraseptal emphysema. An abdominal ultrasound was normal. The results of laboratory tests showed a leukocytosis of 14,930 cells/mm and that hydatid serology tests (hemagglutination, bentonite flocculation and latex agglutination tests) were positive.

**Figure 3:**
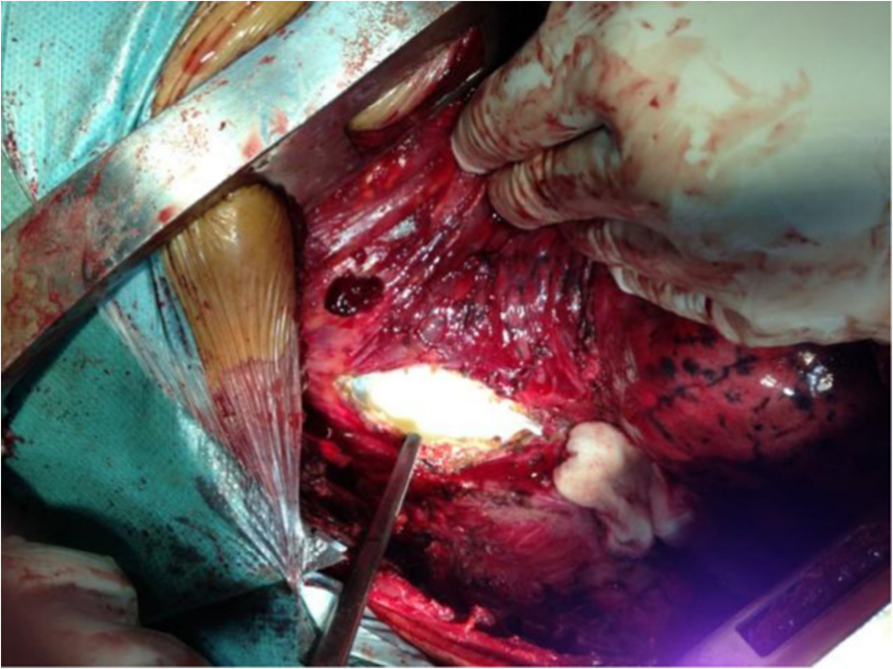
Image taken intraoperatively objectifying the pericyst and cyst wall (white).

**Figure 4:**
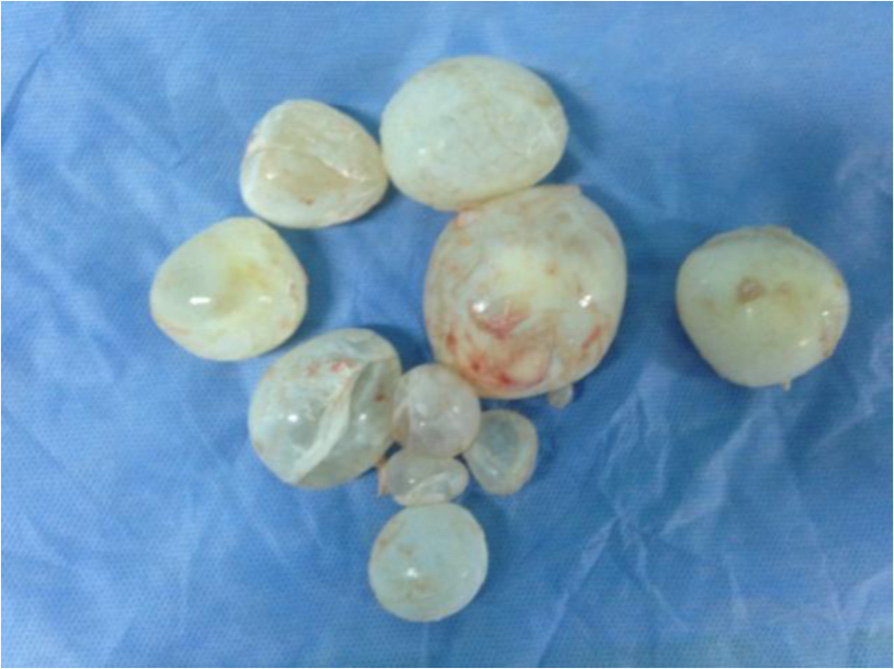
Daughter vesicles.

He underwent a posterolateral thoracotomy through the right-side sixth intercostal space, showing a posterior mediastinal cystic mass that occupied the upper and anterior mediastinum. After a needle aspiration of the cyst, several vesicles were removed (Figures 3 and 4). Extensive washing of his mediastinal cavity was performed with hydrogen peroxide. The postoperative outcome was adequate. He was treated with albendazole 400mg one tablet per day. His condition evolved favorably.

## Discussion

Hydatid disease is a parasitic disease secondary to the development of the larval form of *E. granulosus*. Humans can be accidentally infested by ingesting food contaminated with parasite eggs or by direct contact with a sick dog.

On entering the intestines of humans, the parasite gains further entry to locate itself in the liver or lung, and its passage into the systemic circulation is responsible for its location in diverse locations [1]. These two organs are a blood filter for the dissemination of the parasite, thus explaining the rarity of hydatid in all other locations [2]. Primitive mediastinal localization is one of the rarest. Approximately 100 cases of the above have been published in the English literature [2]. In endemic countries, the incidence of mediastinal hydatidosis varies between 0.5% [3] and 2.6% [2] of all chest locations.

The pathogenesis of mediastinal localization of HC remains controversial [1]. Some findings argue in favor of the hypothesis of fissuring an hydatid liver or lung into the systemic circulation, allowing the parasite to settle in the mediastinum [1,4]. Mediastinal localization could also come from transdiaphragmatic dissemination or via lymphatic abdominal hydatidosis [4]. In our case, postoperative dissemination in the mediastinum from the pulmonary cyst is the most plausible mechanism.

HC of the mediastinum is often revealed by chest pain and signs of mediastinal compression (dyspnea, dysphagia, dysphonia), which can be discovered incidentally or by a complication (rupture in the heart or in a large vessel) [1,5].

Imaging plays a vital role in the diagnosis and staging of lesions. Chest radiography oriented the diagnosis by showing a mediastinal water tone, often rounded or oval. Thoracic ultrasound allows confirmation of the diagnosis when the lesion is accessible. Thoracic ultrasound also reveals the fluid character of the opacity and, in many cases, the proligerous membrane, pathognomonic of HC [3,6]. It also clarifies the univesicular or multivesicular structure characteristic of HC. In the majority of cases, a chest CT can confirm the diagnosis by objectifying a mass of fluid density, often very limited, unmodified by the injection of contrast, however, contrast uptake by a pericyst can be observed [3,6]. The cyst may also include thin walls testifying to its multivesicular character. The presence of peripheral calcifications supports diagnosis. A predilection for the posterior compartment of the mediastinum has been reported in the literature [3,7].

Magnetic resonance imaging (MRI) is indicated in cases of intolerance to iodine or pregnant women. These different ways of cross-sectional imaging (ultrasound, CT, MRI) allow, in most cases, HC mediastinal cystic masses to be differentiated from other mediastinal cystic masses, such as enteric cyst, cystic lymphangioma, pleuropericardial cyst and bronchogenic cyst. The existence of locations in diaphragmatic hydatid advocates the hydatid nature of the mediastinal liquid mass. However, in case of doubt, it is only in the intraoperative period that the diagnosis of mediastinal HC is supported [3]. Hydatid serology is the only biologic aid to preoperative diagnosis. Its negativity does not exclude the diagnosis, but poses a diagnostic and therapeutic problem [4]. The hemagglutination, bentonite flocculation and latex agglutination tests are the procedures of choice at present. The fluorescent antibody test shows much promise, but requires further evaluation [6].

Surgical treatment of mediastinal HC is essential. It consists of a cystectomy associated with total or partial pericystectomy. The surgical approach is according to cases, a posterolateral thoracotomy, anterolateral thoracotomy or median sternotomy [3,4,7]. The postoperative course is classically simple with no mortality [3,4]. No cases of recurrence have been described. The value of medical treatment based on albendazole remains controversial.

## Conclusions

The mediastinum is an atypical location of hydatid cyst, rare even in endemic countries. Diagnosis is based on radiography, biology (hydatid serology) and histology study. This case report indicates that the etiology of hydatidosis should be kept in mind when a patient presents with signs of mediastinal compression.

## Consent

Written informed consent was obtained from the patient for publication of this case report and accompanying images. A copy of the written consent is available for review by the Editor-in-Chief of this journal.

## Competing interests

The authors declare that they have no competing interests.

## Authors’ contributions

YM was the surgeon responsible and is the first author, OA was the second surgeon, and was a major contributor in writing the manuscript. MK was the surgeon aid. YB analyzed the patient data regarding the serology. NI recruited the patient. YR was the anesthesist reanimator. BF contributed to the writing and corrections of the manuscript. All the authors read and approved the final manuscript.
